# Over-Expression of Copper/Zinc Superoxide Dismutase in the Median Preoptic Nucleus Attenuates Chronic Angiotensin II-Induced Hypertension in the Rat

**DOI:** 10.3390/ijms151222203

**Published:** 2014-12-02

**Authors:** John P. Collister, Mitch Bellrichard, Donna Drebes, David Nahey, Jun Tian, Matthew C. Zimmerman

**Affiliations:** 1 Department of Veterinary and Biomedical Sciences, College of Veterinary Medicine, University of Minnesota, St. Paul, MN 55108, USA; E-Mails: bellr007@umn.edu (M.B.); drebe003@umn.edu (D.D.); nahe0001@umn.edu (D.N.); 2 Department of Cellular and Integrative Physiology, University of Nebraska Medical Center, Omaha, NE 68198, USA; E-Mails: jtian@unmc.edu (J.T.); mczimmerman@unmc.edu (M.C.Z.)

**Keywords:** superoxide dismutase (SOD), median preoptic nucleus, hypertension, angiotensin II, reactive oxygen species, brain, hypothalamus

## Abstract

The brain senses circulating levels of angiotensin II (AngII) via circumventricular organs, such as the subfornical organ (SFO), and is thought to adjust sympathetic nervous system output accordingly via this neuro-hormonal communication. However, the cellular signaling mechanisms involved in these communications remain to be fully understood. Previous lesion studies of either the SFO, or the downstream median preoptic nucleus (MnPO) have shown a diminution of the hypertensive effects of chronic AngII, without providing a clear explanation as to the intracellular signaling pathway(s) involved. Additional studies have reported that over-expressing copper/zinc superoxide dismutase (CuZnSOD), an intracellular superoxide (O_2_·^−^) scavenging enzyme, in the SFO attenuates chronic AngII-induced hypertension. Herein, we tested the hypothesis that overproduction of O_2_·^−^ in the MnPO is an underlying mechanism in the long-term hypertensive effects of chronic AngII. Adenoviral vectors encoding human CuZnSOD (AdCuZnSOD) or control vector (AdEmpty) were injected directly into the MnPO of rats implanted with aortic telemetric transmitters for recording of arterial pressure. After a 3 day control period of saline infusion, rats were intravenously infused with AngII (10 ng/kg/min) for ten days. Rats over-expressing CuZnSOD (*n* = 7) in the MnPO had a blood pressure increase of only 6 ± 2 mmHg after ten days of AngII infusion while blood pressure increased 21 ± 4 mmHg in AdEmpty-infected rats (*n* = 9). These results support the hypothesis that production of O_2_·^−^ in the MnPO contributes to the development of chronic AngII-dependent hypertension.

## 1. Introduction

It is well-established that angiotensin II (AngII) acting centrally in the subfornical organ (SFO) can mediate hypertension via activation of neurons in the downstream median preoptic nucleus (MnPO). A large body of evidence supports the notion that the SFO, a circumventricular organ (CVO) with an incomplete blood brain barrier located on the rostral wall of the third ventricle, mediates many of the central dipsogenic and pressor effects of AngII [1,2,3,4,5]. The MnPO of the lamina terminalis has been shown to connect to the SFO both functionally and anatomically [6,7,8,9,10]. The MnPO receives dense afferent input from the SFO [10,11], and its disruption from the SFO or lesion has been shown to decrease drinking, pressor, and vasopressin secretion responses to AngII [12,13,14,15,16].

With regard to the chronic hypertensive effects of AngII, data from our lab has implicated both the SFO and its downstream nucleus, the MnPO, in mediating hypertension induced by chronically elevated levels of AngII. More specifically, we have shown that lesion of either the SFO or MnPO markedly attenuates the development of hypertension induced by a 10 day peripheral infusion of AngII [17,18,19,20]. In an attempt to better understand a possible mechanism mediating this signaling in the MnPO, we investigated the role of superoxide (O_2_·^−^) in the MnPO during AngII hypertension. Previous studies have shown that over-expression of copper/zinc superoxide dismutase (CuZnSOD), an antioxidant enzyme that specifically scavenges O_2_·^−^, in the SFO by central injection of adenovirus encoding CuZnSOD markedly attenuates hypertension induced by peripheral infusion of AngII [21]. These results were accompanied by a decrease in O_2_·^−^ levels in the SFO. These findings were strikingly similar to our previous results demonstrating a marked attenuation of the chronic hypertensive effects of AngII in animals with lesions of the SFO or MnPO [17,18,19,20].

In the current study, we tested the hypothesis that increased levels of O_2_·^−^ in the MnPO contribute to the elevated blood pressure observed in AngII-induced hypertension. To test this hypothesis, CuZnSOD was over-expressed specifically in the MnPO of rats by direct injection of adenoviral vectors encoding CuZnSOD into both the dorsal and ventral MnPO. Rats were then subjected to a 10 day peripheral infusion of AngII similar to that which we have used in our previous studies. Our results demonstrate that the chronic hypertensive effects of AngII are diminished in rats over-expressing CuZnSOD in the MnPO.

## 2. Results

### 2.1. Adenovirus-Mediated Over-Expression of Copper/Zinc Superoxide Dismutase (CuZnSOD) in the Median Preoptic Nucleus (MnPO)

**Figure 1:**
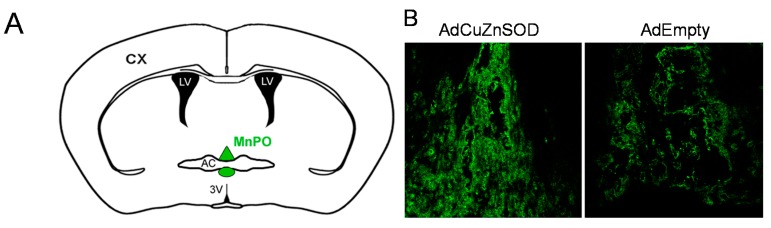
Schematic illustrating a coronal section of the rat hypothalamus (**A**) highlighting both the dorsal and ventral median preoptic nucleus (MnPO) (Green); Representative confocal microscopy immunofluorescent images of hypothalamic coronal sections (**B**) demonstrating copper/zinc superoxide dismutase (CuZnSOD) expression (green fluorescence) in the dorsal MnPO from an AdCuZnSOD-injected (**left**) and an AdEmpty-injected rat (**right**). (MnPO—Median preoptic nucleus; AC—Anterior commissure; 3V—Third ventricle; LV—Lateral ventricle; CX—Cortex).

To confirm adenoviral-mediated over-expression of CuZnSOD in the MnPO, immunofluorescence confocal microscopy was performed on brain sections from rats that received an injection of adenoviral vectors encoding human CuZnSOD (AdCuZnSOD) or control vector (AdEmpty) into the MnPO. As shown in the representative confocal microscopy images (Figure 1B), protein levels of CuZnSOD in the dorsal MnPO approximately 5 weeks after central injection of AdCuZnSOD were markedly elevated compared to endogenous CuZnSOD levels in AdEmpty-injected rats. It should be noted that all AdCuZnSOD rats included in the final hemodynamic analyses (described below) were confirmed to have robust expression of CuZnSOD in and confined to the MnPO relative to the minimal fluorescence detected in the MnPO of AdEmpty-injected rats.

### 2.2. CuZnSOD Over-Expression in the MnPO Attenuates Angiotensin II (AngII)-Induced Hypertension

**Figure 2:**
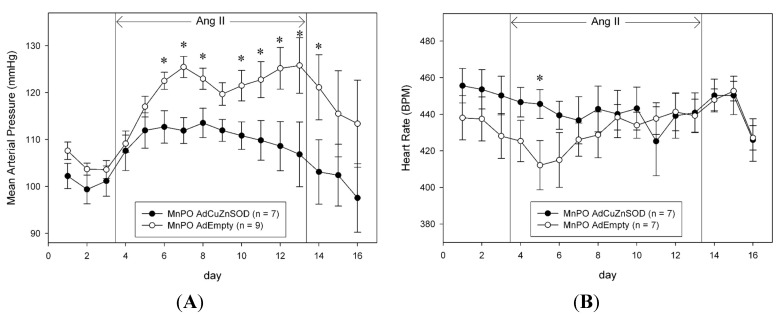
Average 24-h mean arterial pressure (**A**) and heart rate (**B**) recorded during saline infusion (3 days of control and recovery period) and 10 days of AngII infusion (10 ng/kg/min) in rats that were MnPO injected with adenoviral vectors encoding human CuZnSOD (AdCuZnSOD) or control vector (AdEmpty). * *p* < 0.05 * vs.* AdEmpty-injected rats.

As shown in Figure 2A baseline mean arterial pressure (MAP) was not different between AdCuZnSOD- and AdEmpty-injected rats during the control saline infusion period (average during 3 days: 101 ± 3 and 105 ± 2 mmHg, respectively). However, during the peripheral (*i.e.*, intravenous) infusion of AngII, MAP was significantly lower in AdCuZnSOD-treated rats on days 3–5 and 7–10 of AngII treatment and on day 1 of the recovery period compared to AdEmpty-treated rats (Figure 2A). By day 10 of AngII infusion, MAP had reached 126 ± 6 mmHg in AdEmpty-injected rats but was only 107 ± 7 mmHg in AdCuZnSOD rats. Heart rate (HR) (Figure 2B) was not different between AdCuZnSOD and AdEmpty-treated rats during the 3 day control period (average during 3 days: 453 ± 10 and 435 ± 12 beats/min, respectively). HR tended to be decreased in AdCuZnSOD rats throughout the initial days of AngII infusion, and on day 2 of AngII infusion this decrease was statistically significant compared to AdEmpty-injected rats.

### 2.3. CuZnSOD Over-Expression in the MnPO Does not Alter Sodium and Water Balance in AngII Hypertensive Rats

**Figure 3:**
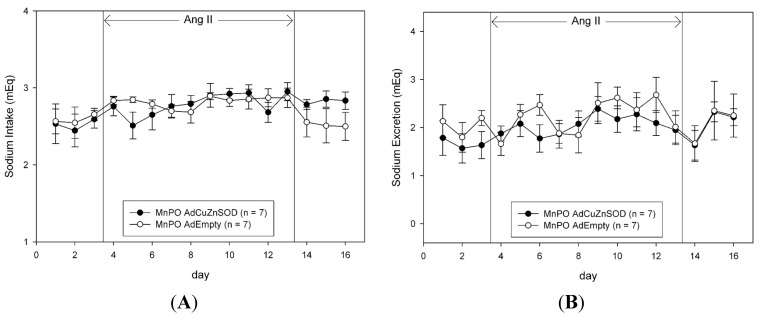

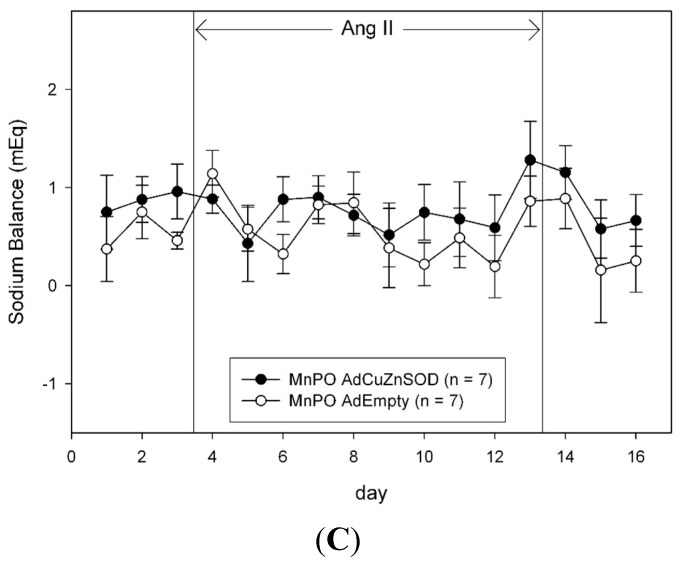
Average 24-h sodium intake (**A**); sodium output (**B**); and sodium balance (**C**) during saline infusion (3 days of control and recovery period) and 10 days of Angiotensin II (AngII) infusion in rats that were MnPO injected with AdCuZnSOD or AdEmpty.

**Figure 4:**
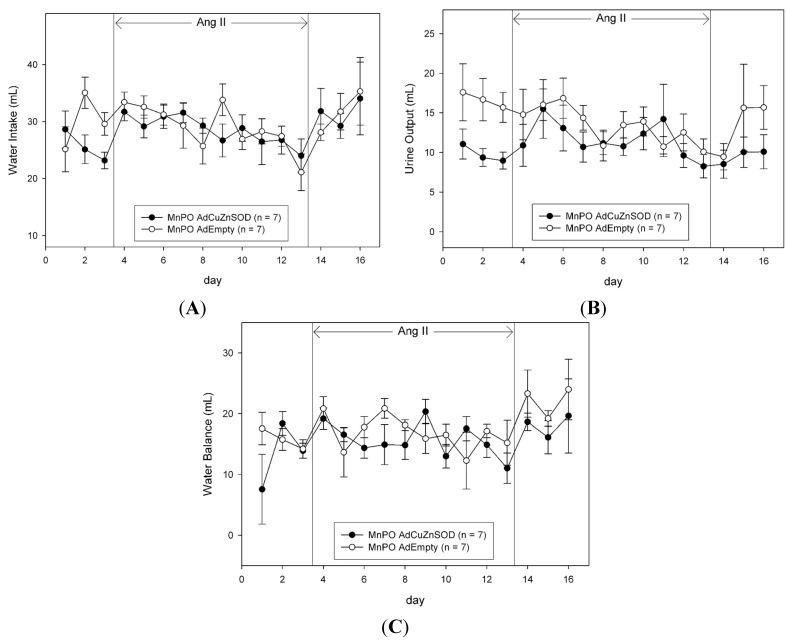
Average 24-h water intake (**A**); urine output (**B**); and water balance (**C**) during saline infusion (3 days of control and recovery period) and 10 days of AngII infusion in rats that were MnPO injected with AdCuZnSOD or AdEmpty.

To determine if CuZnSOD over-expression in the MnPO also alters body fluid homeostasis in AngII hypertension, we measured sodium intake, sodium excretion, and sodium balance (Figure 3), as well as water intake, urine output, and water balance (Figure 4). Unlike the changes in MAP observed in AdCuZnSOD-treated rats (Figure 2), over-expressing CuZnSOD in the MnPO failed to alter sodium and water balance during the entire experimental protocol compared to AdEmpty-injected rats.

## 3. Discussion

Chronic peripheral AngII infusion in experimental animals has frequently been used to produce a slowly developing hypertension that is thought to mimic human hypertension [22,23]. Much research has been conducted to elucidate the neurogenic components of this model of hypertension [24,25]. Although thought to be a useful and reproducible model in this regard, the integration of various cardiovascular control brain regions, pathways, and intracellular signaling mechanisms involved in producing this gradual hypertensive effect of AngII remain to be fully understood. Herein, we report that O_2_·^−^ signaling in the MnPO nucleus of the rat plays an important role in the chronic hypertensive effects of peripherally administered AngII. Our results demonstrate a robust hypertensive effect of approximately 25 mmHg after 10 days of AngII treatment (10 ng/kg/min) in rats that received direct MnPO injections of a control, empty adenovirus vector. This effect was markedly attenuated in rats that were MnPO-injected with AdCuZnSOD prior to the start of AngII infusion. These data support the idea that O_2_·^−^ in the MnPO has a significant role in the signaling mechanism underlying this model of chronic AngII hypertension.

The SFO is one of the most studied CVOs and has been widely implicated as a primary central target for circulating AngII and its dipsogenic and hypertensive effects. As such, in SFO lesion studies, we have previously shown that the SFO is necessary for the full hypertensive response to chronic peripheral AngII infusion [17,18]. Furthermore, it has been reported in mice that this effect is mediated through O_2_·^−^-dependent signaling in the SFO [21]. Downstream of this CVO, the MnPO receives reciprocal inputs from the SFO and other brain regions believed to form the sympathoexcitatory pathway following activation by AngII at the SFO [10,11,26,27]. In an attempt to further determine the role of the MnPO in the chronic effects of AngII, our lab also reported a similar decrease in the long term hypertensive response to AngII in rats with either total ablation of the MnPO or chemical lesions of the MnPO which spare the fibers of passage in this nucleus [19,20]. Considering these previous observations, in the present study we sought to further determine the role of O_2_·^−^ as an intracellular signaling molecule specifically in the MnPO in this model of AngII hypertension. In contrast to previous studies that employed the use of intracerebroventricular (ICV) injections of adenovirus, which results in adenovirus expression throughout the ventricular system, in this study, adenovirus encoding CuZnSOD was specifically injected into the MnPO to directly and selectively over-express this O_2_·^−^ scavenging enzyme in this important cardiovascular control brain region. Our current results demonstrate a strikingly similar inhibition of the development of hypertension during 10 days of intravenous AngII infusion to what we have previously reported in SFO or MnPO lesioned rats.

While the present study supports the role of reactive oxygen species, particularly O_2_·^−^, in the MnPO mediating the chronic hypertensive effects of AngII and these results are similar to the previous study of Zimmerman *et al.* [21] which focused on the SFO, there are several notable differences. In both studies, animals centrally injected with AdCuZnSOD had a marked attenuation of AngII hypertension, although the previous study utilized a mouse model that received AngII subcutaneously via osmotic minipump at a dose of 600 ng/kg/min over 16 days compared to the present study which utilized continuous IV infusion for 10 days at a dose of 10 ng/kg/min. The attenuation of hypertension reported by Zimmerman *et al.* [21] was not noted until day 11 of AngII infusion and the peak MAP observed in control adenovirus-injected animals was 150–160 mmHg. In contrast, in the present study, the peak MAP in AdEmpty-injected animals infused with AngII reached approximately 125 mmHg and over-expression of CuZnSOD in the MnPO significantly attenuated the rise in MAP on days 3–5 and 7–10. These differences are likely attributable to the different dose and route of AngII infusion and to the species (mouse *vs.* rat) used in these studies. Nevertheless, the results are straightforward and similar in that hypertension developed gradually in both groups of control animals during peripheral AngII infusion and this was markedly attenuated after several days in animals over-expressing CuZnSOD; thus, equally implicating O_2_·^−^-dependent signaling in the SFO and the MnPO as a mechanism driving the chronic hypertensive effects of AngII.

Also, as previously noted, another difference between our current study and previous studies is that AdCuZnSOD was delivered by direct injection into the site of interest, namely the MnPO. The previous study by Zimmerman, *et al.* [21] utilized ICV injections of adenovirus to target the SFO non-specifically, although they predominantly observed SOD over-expression in the SFO and thus concluded that AngII-induced hypertension is attenuated by increased scavenging of O_2_·^−^ in the SFO. In the present study, in order to the target the MnPO, which unlike the CVO, is located behind the blood brain barrier, we utilized direct injections into this important cardiovascular control nucleus. Our lab has previous experience in targeting the MnPO via lesion or microinjection [19,20], and therefore posit that this technique was the most adequate to effectively target this nucleus. However, it should be noted that adenovirus vectors can be retrogradely transported along neuronal axons. Therefore, it is possible that neurons projecting from other areas to the MnPO (e.g., the SFO) could have been infected with AdCuZnSOD injected directly into the MnPO. As such, we carefully examined other areas of the brain during our CuZnSOD immunofluorescent confocal microscopy experiments. In a few rats, CuZnSOD over-expression was noted in the SFO, although at a much reduced level than that seen in the MnPO; while the majority of rats included in the analyses had no detectable over-expression of CuZnSOD in the SFO. Nevertheless, we cannot exclude the possibility that minimal over-expression of CuZnSOD in the SFO contributed to the blunted increase in AngII-dependent hypertension in rats MnPO-injected with AdCuZnSOD. An additional potential limitation of the current study is that we did not directly measure O_2_·^−^ levels in the MnPO of AdEmpty- or AdCuZnSOD-injected rats. However, previous studies using the same adenoviral vector to over-express CuZnSOD in the brain clearly demonstrate that the adenovirus-expressed CuZnSOD is active and does decrease O_2_·^−^ levels [21].

## 4. Experimental Section

All methods were approved by the Institutional Animal Care and Use Committee (IACUC protocol number: 1109A04504; approval date: 1 November 2013) at the University of Minnesota and conducted in accordance with institutional and National Institutes of Health guidelines. Male Sprague-Dawley rats (Charles River Laboratory, Wilmington, MA, USA) weighing 250–275 g were used for experiments and housed in an animal housing facility with a 12–12 h light-dark cycle.

### 4.1. Surgical Procedures

Rats were anesthetized with ketamine (75 mg/kg) and xylazine (10 mg/kg), and placed in a stereotaxic apparatus (model No. 900; David Kopf Instruments; Tujunga, CA, USA). A dorsal midline incision was made in the skin of the skull. Bregma and lambda were exposed, repositioned to be on the same horizontal level, and a 2.0 mm hole was drilled into the skull. Replication-deficient recombinant adenoviruses (Ad5-CMV) encoding human CuZnSOD (AdCuZnSOD) or control adenovirus (AdEmpty) were obtained from Viraquest Inc. (North Liberty, IA, USA) and injected into the MnPO of rats (*n* = 9 AdCuZnSOD; *n* = 7 AdEmpty). Titers of viral vectors were pair matched at 10^9^ pfu/mL. A Hamilton syringe was lowered through the midline hole in the skull to the following 2 coordinates (mm caudal and ventral relative to bregma; −0.35, −7.2 and −0.4, −6.1), allowing placement of the injector near both the dorsal and ventral aspects of the MnPO (see Figure 1A). 100 nL of adenovirus was injected at both locations in the MnPO over a period of 5–10 min. The hole in the skull was repaired with bone wax and the skin closed with 3–0 (0.2 mm) silk suture. After surgery, rats were given an injection of the antibiotic gentamicin (2.5 mg, intramuscular) and the analgesic butorphanol tartrate (0.075 mg, subcutaneously).

One week after adenoviral injections, rats were implanted with radiotelemetric pressure transducers (model No. TA11PA-C40, Data Sciences International, St. Paul, MN, USA) and femoral catheters as previously described [17,20] for continuous blood pressure and heart rate monitoring and blood sampling, respectively. Briefly, a midline abdominal incision was made and the descending aorta was exposed. The aorta was clamped and the catheter of the transducer was introduced distal to the clamp and glued in place. The aortic clamp was released, and the transmitter unit was attached to the abdominal wall with 3–0 surgical suture during closure of the abdominal cavity. Next, a small ventral incision was made in the left leg and the femoral vein exposed. The vein was tied off and the catheter introduced approximately 9 mm into the vein and tied in place. The catheter was then tunneled subcutaneously to an exit location between the scapulae and passed through a flexible spring connected to a single-channel hydraulic swivel. After surgery, rats were given an injection of the antibiotic gentamicin (2.5 mg, I.M.) and the analgesic butorphanol tartrate (0.075 mg, S.C.). After transmitter and catheter implantation, rats were placed in metabolic cages, given distilled water and a 0.4% NaCl diet *ad libitum*, and started on a continuous IV isotonic saline infusion of 7 mL/24 h.

### 4.2. Experimental Protocol

After one week of recovery, rats entered the following protocol: 3 days of baseline control, 10 days of intravenous AngII infusion (10 ng·kg^−1^·min^−1^), and 3 days of recovery. AngII was dissolved in sterile 0.9% saline and given at a rate of 7 mL/24 h. During control and recovery periods, all rats received intravenous infusion of normal saline (7 mL/24 h).

The daily food and water intake and urine output were measured gravimetrically. Sodium intake was calculated as the sum of the sodium received from the daily intravenous infusion (1 mmol/day), plus the product of the food intake and the sodium content of the food (2.0% NaCl, 0.35 mmol/g). Urinary sodium concentration was measured with an ion-specific electrode (NOVA-5+; Biomedical, Waltham, MA, USA). The daily urinary sodium excretion was calculated as the product of the urine output and urinary sodium concentration. The daily sodium and water balances were calculated as the difference between intake and urinary excretion of sodium and water, respectively.

At the end of the protocol, all rats were deeply anesthetized and perfused with heparinized saline (20 U/mL; 150 mL) followed by 4.0% paraformaldehyde via the aorta. Brains were removed and transferred to 4.0% paraformaldehyde. Brain expression of CuZnSOD, particularly in the MnPO, was determined by immunofluorescence and confocal microscopy, as previously described [21]. Briefly, brain sections were incubated with CuZnSOD antibody (sheep anti-CuZnSOD; The Binding Site, Birmingham, UK) diluted 1:500 in 2% normal horse serum and 0.3% Triton overnight at 4 °C, washed, and further incubated with donkey anti-sheep AlexaFluor 488 secondary antibody (Invitrogen, Molecular Probes, Carlsbad, CA, USA) diluted 1:200. Sections were washed, mounted on slides, and imaged with confocal microscopy (Zeiss 510 Meta Confocal Laser Scanning Microscope, Carl Zeiss Microscopy GmbH, Jena, Germany).

### 4.3. Statistical Analysis

Data are reported as mean ± SE. One- or two-way ANOVA combined with a Student–Newman–Keuls test was used for comparisons. Differences were considered significant at *p* < 0.05.

## 5. Conclusions

In conclusion, the results of our present study support and extend previous findings, and clearly implicate O_2_·^−^ signaling in the MnPO nucleus as a contributing mechanism driving the development of hypertension in a model of chronic, peripheral AngII infusion. Future studies are warranted to investigate the precise intraneuronal signaling intermediates sensitive to the increase in O_2_·^−^. In addition, our data presented herein support the advancement of new studies designed to develop novel antioxidant-based therapeutics that target the unique cardiovascular control brain regions, such as the MnPO, and specifically scavenge O_2_·^−^ for the improved treatment of hypertension.
